# Undocumented immigrants’ and immigrant women’s access to healthcare services in the Basque Country (Spain)

**DOI:** 10.1080/16549716.2021.1896659

**Published:** 2021-05-12

**Authors:** Iratxe Pérez-Urdiales

**Affiliations:** Department of Nursing, Iund University of the Basque Country (UPV/EHU), Biscay, Spain

**Keywords:** Undocumented immigrants, immigrant women, health service accessibility, free clinic, right to health

## Abstract

**Background:**

Immigrant populations experience diverse barriers to access healthcare services in the host countries. Among them, undocumented immigrants have more restricted legal access conditions and higher risk of having poorer health. Likewise, women are more likely to seek healthcare and face gender-based factors that hinder their access.

**Objective:**

This thesis analysed the access of undocumented immigrants and immigrant women to public healthcare services in the Basque Country (Spain).

**Methods:**

The thesis contained three sub-studies, carried out with qualitative and quantitative methods. For the quantitative approach, the trend in the number of consultations in a free clinic for undocumented immigrants was analysed before and after the launch of a new law, using a negative binomial regression analysis (n = 9,272). For the qualitative approach, qualitative content analysis was applied to 25 in-depth interviews with 14 immigrant women and 11 free clinic healthcare professionals.

**Results:**

No clear relationship was found between the application of more restrictive legal conditions for immigrants to access public healthcare services and the trend of attendance of undocumented immigrants to a free clinic. Access of undocumented immigrants and immigrant women to healthcare services was subject to barriers dependent on their characteristics, health system functioning, legal requirements and a stereotyped and poor social consideration of immigrants, shared by professionals at the health centres. Meanwhile, provision of legal information and support by individual professionals, social organizations and personal networks represented main facilitators for accessing.

**Conclusions:**

For the access of undocumented immigrants and immigrant women, structural and individual barriers based on their social vulnerability were found. Among others, gender-based violence reduced women’s possibility to access healthcare services and being undocumented led to restricted access entitlement and to fear rejection at health centres. Therefore, besides ensuring immigrants’ legal entitlement, there is need of promoting rights-based attention to get more inclusive health systems.

## Background

The doctoral thesis entitled ‘*Does the health system respect immigrants’ right to health? Analysing immigrant women’s healthcare access in the Basque Country’* [1] was a research project on the access of undocumented immigrants and immigrant women to public healthcare services. The thesis considered available quantitative data of free clinics and the experiences and perceptions of immigrants and healthcare professionals working at the free clinics to explore the access to appropriate healthcare services for immigrants in the Basque Country. It was mainly focused on the access of undocumented immigrants and immigrant women from low-income countries, since they are the ones in most vulnerable situations.

In recent years, the number of immigrants in the European Union (EU) has consistently increased [2]. In Spain, by January 2019, registered immigrants were 10.7% of the population [3]. In the Basque Country, which is one of the autonomous regions of Spain, foreign origin persons represented 7.4% of the whole population; having a similar distribution of men and women [4]. Anyway, no official figures exist about how many undocumented immigrants could be residing in Spain or in the regions, which represents a challenge for healthcare providers and policymakers [5].

Studies regarding access of immigrants to healthcare services in Spain have concluded that they utilize them to a lesser extent than natives. In general, the immigrant population used less specialised, primary, and mental healthcare services in comparison to natives, even if they used the emergency services more [6–8]. Among the reasons for immigrants having difficulties accessing healthcare services, are legal restrictions, health systems’ excessive bureaucracy, professionals’ negative attitudes towards immigrants and racism [9,10]. Restrictive legislation is often represented as the greatest barrier for accessing healthcare services for certain groups of immigrants, namely undocumented ones and asylum-seekers [9,11,12]. However, access to healthcare is dependent on many individual and systemic factors, as different subgroups of populations may have a very different healthcare access experience, even under the same level of entitlement.

In the European context, undocumented immigrants, which are the non-nationals who enter or stay in a country without the appropriate legal documentation [13], have a more restricted access to healthcare services and higher risk of having poorer physical and mental health [14–16]. Further than the legal restriction to access healthcare services, due to their social vulnerability and poorer living conditions, barriers like lack of awareness about their entitlement, fear of being reported to immigration authorities or poor local language skills also hinder their access [9,14,16,17]. Furthermore, among immigrants, women are more likely to present mental and reproductive health issues than their male counterparts [18,19]. In addition, women are more frequently exposed to gender-based violence during their migration process in its multiple forms [20].

### Access to national and regional health systems in Spain

In Spain, access to the National Health System, which include all regional health systems, is ruled by the centralised state legislation and it is known for offering broad services to all immigrants, being close to the universal healthcare access [21]. In addition, each regional health system complements the provision of healthcare with services stated in regional legal norms. Access to all healthcare services is bound to council registration, which allows for getting the personal healthcare card, the document that entitles individuals to healthcare access [22].

In 2012, using the economic crisis as justification, the Spanish Government enacted a law that dramatically reduced access to healthcare services for undocumented immigrants to the National Health System. Consequently, in 2013, the regional Basque Government, the region where data were collected, launched the Basque Decree 114/2012 to reduce the negative impact of the new Spanish law on immigrants’ access. Even then, the conditions for all immigrants to access healthcare services in the regional health system became more restrictive. It was during this period that the studies were conducted. In July 2018, due to a government change, the access conditions from before 2012 were restored in the whole National Health System, including the Basque Country.

For providing attention to those excluded from public health systems, who are mainly undocumented immigrants, in some countries free clinics exist, which are usually run by non-governmental organisations (NGOs) [14,16,23–25]. A free health clinic is defined as ‘a private, non-for-profit, community-based organization that offers services such as primary and secondary medical and dental care […] These services are offered for no cost or a small fee to low-income, uninsured or underinsured people’ [25]. In the Basque Country, besides the regional public health system, two types of free clinics exist: one providing primary healthcare and social services to those immigrants who do not fulfill the criteria to access the public healthcare services, who are all undocumented immigrants except a few cases, and three providing sexual and reproductive healthcare services to local and immigrant women, regardless of their legal status.

### Conceptual framework

Access to healthcare acts as an important health determinant [26] and is central in the performance of health systems. In the thesis, access was considered as the opportunity to reach and obtain appropriate healthcare services in situations of perceived need for care [27]. In order to describe in a more comprehensive way the different aspects that access may entail, the interrelations of a multi-level model of access, a patient-centred access framework and the right to health model were used (Table 1).

**Table 1.** Summary of the conceptual frameworks guiding the thesis

Multi-level access to healthcare	Person-centred access to healthcare	Right to health
*Entitlement level* Written, formal instruments that have a direct bearing on the available healthcare services for immigrants. Conventions, charters and national or regional laws and policies		*Entitlement* to receiving healthcare as timely and appropriate medical care and underlying determinants of health Obligations of social actors on the right to health Guiding principles of the right to health
*Access level* The gateways to reach healthcare and the extent to which migrants are able to receive the healthcare services that they are entitled to Related to the factors that inhibit or facilitate the realization of their entitlement	*Dimensions of accessibility and related abilities*: Approachability. The extent the health services and the way to reach them can be identified. Related to the ability to perceive need for care Acceptability. Cultural and social factors that make persons to accept different aspects of the service. Related to the ability to seek care Availability. Physical existence of the healthcare resources with sufficient capacity to produce services and being reachable in a timely manner. Related to the ability to reach healthcare Affordability. Economic capacity for people to spend resources and time on accessing healthcare. Related to the ability to pay and generate economic resources Appropriateness. Fit between services and patients’ needs, given the technical and interpersonal quality of the services. Related to the ability to engage in healthcare	*Characteristics of health systems to fulfil the right to health*: Available. Sufficient in quantity Accessible physically, financially and based on non-discrimination Of good quality. Scientifically and medically appropriate Acceptable. Respect of ethics and being gender sensitive and culturally appropriate
*Appropriateness level* Healthcare services’ ability to integrate an effective assessment of health needs of immigrants Related to the content of the received healthcare services and the extent they are responsive to the particular needs of immigrants		

The first framework, Watters’ multi-level model of access, integrates the levels for a comprehensive examination of access to healthcare services for immigrants and populations in situation of vulnerability. The framework was used as a research roadmap, allowing the exploration of different levels of access, namely 1) entitlement, 2) access, and 3) appropriateness [28]. However, as this model did not define the specific characteristics of each level, Levesque’s patient-centred access to healthcare framework was also utilised.

Levesque’s framework allows the researcher to address and contextualize the experiences of access from the perspective of care-users [27]. It considers the different dimensions of access, which are focused on health systems’ characteristics and how each of them connect with more individual ability to access healthcare: the dimensions of approachability, acceptability, availability, affordability and appropriateness and the, respectively, related abilities to perceive, seek, reach, pay and engage healthcare [27]. However, as this model is centred in the individual experience, it does not consider the entitlement as a crucial aspect for access. That is why, the right to health approach was chosen in order to comprehensively state what the entitlement level of access entails.

The right to health approach is centred on the concept of entitlement and it was useful to understand and explore the roles and responsibilities of the different social actors on its fulfillment [29,30]. Under this paradigm, individuals are conceptualized as right holders while states are responsible for ensuring the highest attainable standards of health to individuals. Even if the right to health includes different factors or conditions that protect and promote health beyond healthcare services [29,30], in the thesis the focus was put on the right to appropriate and timely relevant healthcare, which requires available, accessible, of good quality and acceptable health systems [31].

### Objectives

The main objective of this thesis was to analyse the access of immigrant women and undocumented immigrants to public healthcare services in the Basque Country. Based on the hypothesis that for undocumented immigrants, more difficulty in accessing public health systems means more use of the available free clinics, the first specific objective was stablished: 1) To assess the impact of the implementation of more restrictive conditions for accessing public healthcare services for immigrants on the number of consultations of undocumented immigrants at a primary healthcare free clinic. In addition, in order to get a more complete perspective of the barriers that hinder the access to healthcare services for immigrant women and undocumented immigrants in our setting, exploratory specific objectives were stablished: 2) To determine the perception of healthcare professionals working in free clinics on the barriers and facilitators of access to healthcare services by immigrant women and undocumented immigrants in the Basque Country and 3) To analyse Sub-Saharan African immigrant women’s perceptions and experiences of access to appropriate healthcare services in the Basque Country.

## Methods

Three individual studies formed this doctoral thesis. The methodological approach used for responding to the first objective was quantitative (Part I), while a qualitative approach was used for achieving the second and third objectives (Part II) (Table 2). The use of both approaches was motivated by the purpose of complementing the available numbers of consultations of a free clinic for undocumented immigrants (quantitative component) with the perspectives of healthcare professionals about the access of undocumented and female immigrants and the experiences of access of immigrant women to the Basque public health system (qualitative component).

**Table 2.** Summary of the aims and methods of the three sub-studies included in the doctoral thesis

	Part I	Part II	
Objective	To assess the impact of the implementation of more restrictive conditions for accessing public healthcare services for immigrants stated in the new Basque Decree 114/2012 on the number of consultations of undocumented immigrants at a primary healthcare free clinic	To determine the perception of healthcare professionals working in free clinics on the barriers and facilitators in the access by immigrant women and undocumented immigrants to healthcare services in the Basque Country	To analyse Sub-Saharan African immigrant women’s perceptions and experiences on access to appropriate healthcare services in the Basque Country
Sample andAnalysis	Number of healthcare consultations (n = 9272) from Jan 2007 to July 2017 (intervention on 1^st^ trimester of 2013) Segmented time series. Negative binomial regression adjusted for seasonality, % of registered immigrants and unemployment rate	11 individual interviews with healthcare professionals working in free clinics in the Basque Country Qualitative content analysis	14 interviews with women from 8 Sub-Saharan African countries who have used healthcare services in the Basque Country Qualitative content analysis

### Part I. Quantitative study

In 2013, the new Basque Decree 114/2012 established more restrictive conditions for accessing public healthcare services for immigrants, which mainly affected the undocumented ones. Based on the hypothesis that more difficulty in accessing public healthcare services led to more use of the available free clinics, a segmented time series approach was used to analyse the trends on the number of consultations of undocumented immigrants in a primary healthcare free clinic, before and after the law launch.

The application of the Basque Decree 114/2012 in January 2013 was considered the intervention point, so the intervention phase was defined as the first quarter of 2013. Therefore, the pre-intervention phase was settled from January 2007 to December 2012 and the post-intervention phase from January 2013 to June 2017.

First, the number of consultations was counted manually from registration forms completed during the health consultations, extracted into an Excel spreadsheet on a quarterly basis and exported to Stata 13.0 software for analysis. Frequencies for total and new patient consultations, country of origin and diagnosis for men and women were collected from the free clinic database for descriptive analysis (Table 3).

**Table 3.** Characteristics and diagnoses of the patients at the free clinic (January 2007 to June 2017)*

	MEN	WOMEN
Total	7134 (76.94%)	2138 (23.06%)
Attending first time	40.03%	49.58%
REGIONS		
Latin-America	7.04%	56.55%
North Africa	54.34%	18.06%
Eastern Europe	3.22%	7.32%
Asia	2.65%	0.57%
Rest of the world	1.67%	0.49%
DIAGNOSES		
Respiratory system	18.43%	10.10%
Digestive system	14.31%	10.16%
Tegumentary system	13.08%	6. 57%
Musculoskeletal system	14.68%	10.53%
Genitourinary system	2.90%	14.73%
Others	36.60%	47.90%

*Data was only available for new patients and those patients who maintained the same diagnose as in the previous consultation, representing a total of 4,707 people

The data on the number of consultations were adjusted for Biscay province’s unemployment rate, percentage of registered immigrant population and seasonality. Unemployment rate was used to capture the possible influence of the financial crisis on the number of immigrants attending the free clinic. The percentage of registered immigrant population aimed to capture the variability in the amount of immigrants in the province. Finally, the seasonality captured the summer holiday period (third quarter of each year), when the number of consultation days clearly decreased every year.

Data were represented as the following equation:

where Y represented the outcome variable (number of healthcare consultations); ß0 was the baseline level of the outcome at the beginning of the period; ß1 estimated the structural trend in the outcome before the intervention; ß2 estimated the immediate impact of the intervention through the change in level of the outcome and ß3 reflected the change in the trend of the outcome after the intervention.

Negative binomial regression analysis was applied to the data, obtaining rate ratios (RR) and the 95% confidence intervals (95% CI), stratified by sex.

### Part II. Qualitative studies

For exploring the perceptions and experiences on immigrant women’s and undocumented immigrants’ access to healthcare services, in-depth interviews were conducted with healthcare professionals and immigrant women. Clearance from the university ethical committee was obtained before contacting any participant, who gave oral and written consent before starting the interviews.

#### Perception of healthcare professionals

These participants were purposively selected for having a deep knowledge of the immigrants’ social and healthcare situation, due to their experience of providing healthcare to undocumented immigrants and immigrant women who are not legally entitled to public healthcare services.

For data collection, the responsible persons of the four free clinics of the Basque Country were contacted. The study was presented to them so they could offer to the healthcare professionals the possibility to participate. The ones who agreed to take part contacted the researcher in order to receive further information and to fix a date and an adequate place for the interview. Data collection took place between September and December 2015. Eleven interviews with healthcare professionals were carried out.

A semi-structured interview guide was used to conduct the interviews. After collecting some socio-demographic data from each informant (Table 4), questions regarding their experiences of the attention of immigrants and the barriers of their access based on the laws, the institutional practices, being undocumented and immigrants’ personal characteristics were posed. The guide was modified in order to cover important issues that emerged during the data collection process, like the influence of nationality, and of the exposition to gender-based violence in the access of immigrant women to healthcare services. Data collection continued until no remarkable additional information related to the research question seemed to appear. Interviews lasted from 35 to 70 minutes and were carried out in Spanish.

**Table 4.** Socio-demographic characteristics of the participants of qualitative studies (Part II)

Healthcare professionals´ characteristics						
	Primary health attention free clinic		Sexual and reproductive health free clinics			
			Centre 1	Centre 2		Centre 3
Occupation	(4) physician (1) nurse (1) midwife		(1) nurse (1) psychologist	(1) physician (1) nurse		(1) physician
Works/Has worked in the public healthcare system	(4) physician (1) nurse (1) midwife			(1) physician		(1) physician
Years of experience in the free clinic	(3) +10 (1) 2 (1) 4 (1) 9		(2) +10	(1) 1 (1) +10		(1) 8
Immigrants women´s characteristics						
Migration status		Documented (10) of which have been undocumented before (4)			Undocumented (4)	
Country of origin		Cameroon Guinea Bissau (2) Senegal Angola (3) Central African Republic Democratic Republic of Congo Gambia			Democratic Republic of Congo (2) Nigeria Cameroon	
Type of residence permit		Regrouped by husband (4) Asylum-seeker (2) Others (4)			None (4)	
Use of Public Health System		Whenever it was needed			1 time (1) 2 times (2) 3 times (1)	
Use of other health services		Free clinic before having Health Card (2) Private health services (1)			Free clinic (4)	
Possession of Health Card		10			None (4)	
Age		25 to 35 (3) 36 to 60 (6) More than 60 (1)			25 to 35 (3) 36 to 60 (1)	
Language used in the interview		Spanish (9) French (1)			French (2) English (1) Swahili (with translator) (1)	
Time in the Basque Country by the day of the interview		Less than 6 months (1) 6 months-1 year (1) 1–5 years (2) More than 5 years (6)			Less than 6 months (2) 6 months-1 year (2) 1–5 years More than 5 years	
Job		Employed (6)			None (4)	

Data were analysed using qualitative content analysis. Graneheim and Lundman’s approach was used to develop the analysis as they propose [32]. First, all the interviews were transcribed verbatim. The transcriptions were then imported to the Open Code 4.03 Qualitative Data Analysis [33] software for managing the coding process. In this study, only manifest content was analysed. The analysis started with the careful reading of the text. Then, meaning units, the parts of the text related to the research question, were identified and reduced creating condensed meaning units. These were labelled creating codes. Codes were finally examined and grouped into four categories.

#### Perception and experiences of immigrant women

Based on the results of the previous study, which identified black African immigrant women as the most vulnerable in accessing public healthcare services, 14 immigrant women from eight Sub-Saharan African countries were interviewed. All of them were living in the Basque Country and had used the public healthcare services at least one time by the moment of the interview. Some socio-demographic data were collected from each participant (Table 4). The recruitment was made in the waiting room of the primary healthcare free clinic and among users and workers of different social organizations working with immigrants that fulfilled the inclusion criteria.

The interviews were conducted from June 2016 to October 2017. A semi-structured guide was used, which contained questions regarding the knowledge of the legal norms, the influence of the language, and being undocumented in the access to healthcare services. Other relevant emerging issues were also discussed during subsequent interviews, like the influence of being black or of the racism of the professionals at the health centres in their access. Interviews lasted between 25 and 70 minutes. Nine interviews were conducted in Spanish, one in English, three in French and one in Swahili and French. On three occasions, based on the preference of the participants, translators provided by them were used.

The interviews held in English and Spanish were transcribed verbatim, while the interviews conducted in French were translated into Spanish for analysis. All transcripts were reviewed for accuracy and entered into Open Code 4.03 Qualitative Data Analysis [33] software to organize and code the data. Qualitative content analysis [32] was also the approach used to analyse the interviews with immigrant women, which were coded and finally, all codes were organized forming three categories.

## Results

The main results of the thesis for achieving the specified objectives were the following:

### Part I. Quantitative study

From January 2007 to June 2017, a total of 9,272 health consultations were attended in the primary healthcare free clinic, 76.94% for men and 23.06% for women. 49.58% of the women and 40.03% of the men attended the clinic for the first time (Table 3).

**Figure 1.**
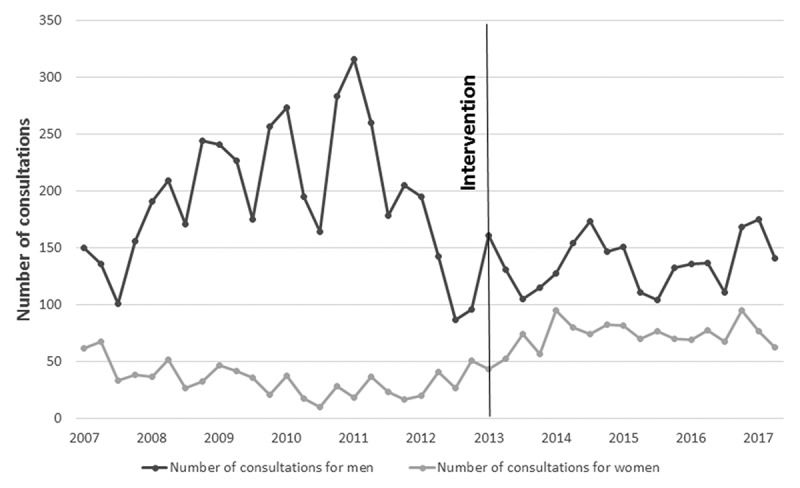
Number of consultations for men and women at CASSIN from January 2007 to June 2017

The total number of consultations for men in the whole period was 7,134, almost 4 times that of women, which was 2,138. The baseline level of the number of consultations for men was 193.23 at the beginning of the whole period, while it was 44.92 for women. During the pre-intervention phase, while a non-significant increase of 2% in the trend of consultations was observed among men from one quarter to the next (RR = 1.02; 95% CI = 0.99, 1.04) there was a significant trend decrease of 2% in consultations for women during the same period (RR = 0.98; 95% CI = 0.95, 1.01). The level of the number of consultations decreased significantly by 40% for men and increased 187% for women per quarter just after the intervention. Finally, the trend of consultations experienced a non-significant increase of 1% per quarter in the post-intervention phase (RR = 1.01; 95% CI = 0.99, 1.03) for men and a statistically non-significant decrease of 1% per quarter in the attendance trend for women (RR = 0.99; 95% CI = 0.96, 1.04) (Table 5) (Figure 1)

**Table 5.** Segmented negative binomial regression results expressed as rate ratios (95% CI in brackets) of the number of consultations at a free clinic by sex (January 2007 to June 2017)

	Baseline level	Pre-intervention	Intervention	Post-intervention
Men	193.23 (147.58, 253.01)	0.99 (0.98, 1.02)	0.65 (0.46, 0.91)	1.01 (0.99, 1.02)
	205.15 (130.37, 322.87)^a^	1.02 (0.99, 1.04) ^a^	0.60 (0.42, 0.84) ^a^	1.01 (0.99, 1.03) ^a^
Women	44.92 (34.35, 58.74)	0.98 (0.96, 1.00)	2.64 (1.78, 3.91)	1.00 (0.99, 1.02)
	56.99 (30.18, 107.64)^a^	0.98 (0.95, 1.01) ^a^	2.87 (1.80, 4.58) ^a^	0.99 (0.96, 1.04) ^a^

^a^Adjusted for unemployment, seasonality and percentage of documented migrant population

### Part II. Qualitative studies

#### Perception of healthcare professionals

After analysing the discourse of healthcare professionals working at free clinics about the access of immigrant women and undocumented immigrants to public healthcare services, four categories were developed: 1) *Tell me who you are so I can tell you how you access*, related to the influence of the personal characteristics of the immigrants on the access; 2) *Once they get the consultation, everything is fine. The problem is with administrative staff*, explained the importance of the attitude of the staff at the health centres; 3) *An inflexible and passive health system* represented the impact of the functioning of the health system and the last category 4) *If you do not fulfil the requirements, you are just out. The law is the law* was related to the effect of health policies in their access.

##### Tell me who you are so I can tell you how you access

Participants considered that women’s approachability and ability to identify healthcare services depended on their origin. Latin-American women in contrast to Africans, besides sharing the language with natives, were considered to have better knowledge on their rights and the functioning on the health system, thus better access.

*Latin women have no problem to access. Africans no way! First, no one is going to listen to them when they speak English or Nigerian … so they will not even pass the counter to reach the healthcare consultation (Nurse)*

Immigrant, but also native women, were considered to be less able to seek care when they were victims of gender-based violence. First, because perpetrators could directly deny their attending healthcare services. Second, because the women themselves could hesitate contacting with healthcare professionals in order to hide their situation of violence.

##### Once they get the consultation, everything is fine. The problem is with administrative staff

Participants described administrative professionals as the main barrier for immigrants’ access due to the poorer attention they provide to immigrants and their lack of knowledge of immigrants’ access rights. In contrast, healthcare professionals’ attention to immigrants was considered more adequate, even if in general, all professionals’ willingness to attend to and fulfil immigrants’ needs was considered weak.

*Attention to immigrants is different than to locals. The staff at the health centres have a huge workload and they are used to always following the same routine. The routine change can create tension so they behave differently with immigrants (Physician)*

##### An inflexible and passive health system

Participants described that information about access to healthcare was not widespread in the community but confined within the health centres. Consequently, it was difficult for populations in situations of vulnerability to be able to seek care that responded to their needs.

*Undocumented immigrants are left out of the health system. But there are some other vulnerable groups, like women working in prostitution or victims of human trafficking, which should be reached in a more direct manner, because they do not attend the consultations themselves even if they need it (Nurse)*

When receiving healthcare, the scarcity of personnel and short consultation times with each patient were considered to reduce the ability of immigrant women to become involved in decision-making with regards to their own health and treatment.

##### If you do not fulfil the requirements, you are just out. The law is the law

All the participants described the legal conditions as the main barrier for immigrants to access the public healthcare services, amplified with their lack of knowledge of their rights. Some participants identified that the legal access conditions, bound to council registration, are difficult to fulfil for immigrants, especially for undocumented ones.

*When they get there, they are asked for the council registration or for money. If they don’t have them, they directly leave without been attended (Physician)*

Moreover, participants considered that undocumented immigrants fear approaching healthcare centres for the potential legal consequences of being identified as undocumented in a public institution, such as being deported.

As facilitating factors, being in contact with social organizations and having a broad personal network were considered a source of information and support for accessing healthcare services. Likewise, finding sensitized professionals with immigrants’ social vulnerability was considered a key facilitator for improving access of this group, including those who are not legally entitled.

#### Perception and experiences of immigrant women

Immigrant women from Sub-Saharan Africa were identified by healthcare professionals working at the free clinics as facing the largest barriers to access the public healthcare services. That directed the focus of the last study on this specific group. After analysing the interviews with Sub-Saharan African immigrant women, the three categories *Fear of entering a health system perceived as not friendly for immigrants; Being attended on professionals’ own communication terms* and *Is mistreatment based on racism or merely on bad luck*?, which were, respectively, related to access conditions, communications with professionals and racism were developed.

##### Fear of entering a health system perceived as not friendly for immigrants

Participants reported that lack of knowledge on the laws regulating access for immigrants, which were considered difficult to accomplish, made them refrain from trying to access healthcare. In contrast, having the healthcare card was considered key for making rights go into effect, despite other barriers.

Undocumented immigrants were considered to avoid seeking care for fearing being reported to state immigration institutions or incurring debt with a public institution, which could result in their deportation. Therefore, being asked to pay for a healthcare consultation, acted as a discouraging factor in continuing asking for healthcare.

*Imagine: I was very sick, but they were not attending me. The man there (administrative professional) told me: “Sorry, we can’t do anything”. He also told me that I could pay and access, but I told him: “To pay? I have no money, no aid, I have nothing”. It was difficult. So I left (Arjana)*

However, attending NGOs to get proper information, reaching health centres accompanied by someone or finding sensitive professionals acted as facilitating factors for access.

##### Being attended on professionals’ own communication terms

Language was experienced as one of the most important barriers by the participants. Lack of communication with administrative staff led to more difficulties on meeting a healthcare professional. Likewise, lack of communication with healthcare professionals hindered their possibility to be diagnosed, being offered a proper treatment or it led to mistakes in the medical treatment.

In addition, those who do not speak the local language perceived that the lack of communication put them in a disadvantaged position, reinforced by a poor consideration about immigrants of certain staff at health centres.

*Ahhh … is very difficult, because a lot of people there (health centre) they don’t speak English. The only thing is to say … slowly … and then I try to understand. I use my phone to translate. Look, it is like … I don’t need to attend you. They tell me: “You are in the Basque Country; you need to speak in Spanish” (Jessy)*

To overcome these situations, staff and immigrants developed strategies to communicate, including the use of translators.

##### Is mistreatment based on racism or merely on bad luck?

Participants perceived that the administrative staff at the health centres behaved differently with them than with natives. Even if healthcare professionals’ vocation was considered incompatible with being racist, some situations of differentiated treatment and mistreatment were also reported. For example, some cases on which a stereotype of immigrants influenced the diagnoses were presented.

Participants working at social organizations connected the poorer attention given to immigrants and black people in the health system with colonial domination of high-income countries. Likewise, they pointed out that usually the staff at the health centres considered immigrants undeserving to receive healthcare.

*All those labels that the history has accumulated on us influence the way we are attended. A social rejection image follows us wherever we go and hinders our access to the health system (Elizabeth)*

In contrast, while some participants connected of the differentiated treatment they received with a systemic tendency of racism, others based it on more individual factors, like individual professionals’ bad day or lack of patience.

## Discussion

The discussion section aims to compare the main findings of the quantitative and qualitative studies with the literature, under a common frame of the three conceptual frameworks. Although results are divided following the Watters’ multi-level model of access, there are close connections as well as overlapping areas between the different aspects of access that each of the levels entail.

The results of the studies indicate that there is a considerable number of barriers and some facilitators in the access of immigrant women and undocumented immigrants to public healthcare services in the Basque Country. Main barriers at entitlement level included lack of entitlement, lack of knowledge on the rights to access healthcare services and difficulties for fulfilling the legal access conditions. At access level, factors such as lack of information, lack of an effective communication with staff at the health centres and fear to be rejected or billed discouraged immigrants from approaching the health centres, especially those in situations of vulnerability, like undocumented immigrants or women exposed to gender-based violence. At appropriateness level, staff’s lack of cross-cultural knowledge and lack of willingness to attend immigrants and racism represented great barriers on the compliance of the health needs of immigrants. For counteracting these barriers, facilitators such as being informed, accompanied or having support by individual professionals, social organizations, free clinics and personal networks were highlighted.

### Entitlement

Considering the right to health approach, states’ core obligation is taking appropriate measures to provide healthcare to vulnerable members of society [29,30]. However, in most European countries, a gap exists between the principles of the right to health and the entitlement to healthcare for undocumented immigrants [12].

Spain, before the law change in 2012 and nowadays, after the new law launched in July 2018, is among the EU countries offering broader services to immigrants [21]. However, the law change in 2012 went clearly against the principle of non-discrimination, as undocumented immigrants were left out of the health system, except in case of emergency, pregnancy or being a minor [29]. Limiting primary healthcare attention increases the demand for emergency care, which is more costly and entails higher risks for public health [34].

In addition, the law change generated confusion for both patients and professionals in relation to the entitlement of immigrants to healthcare [12,15]. Professionals’ lack of awareness was reflected in the increase of the number of entitlement violations reported by social organizations, which reached almost 5,000 cases in the 5 years after the approval of the law [35]. In our results, because of the absence of demographic data on the number of undocumented immigrants residing in the region, the influence of increasing legal restrictions on the access to healthcare services, could not be related to a higher number of consultations of undocumented immigrants in a primary healthcare free clinic.

However, very different trends of attendance of undocumented immigrant men and women were observed over a 10 year period in the same free clinic, as men represented 76.94% of total consultations. Due to the lack of the above-mentioned key demographic information, demographic reasons which could explain these trends could also not be contrasted. Moreover, nor did the proportion of documented registered immigrants shed light on the reasons for the excess number of consultations of men, as from 2006 to 2019, the proportion of sexes in the council register was close to 50% each [4]. An explanation regarding the lower number of female patients arriving at the free clinic could be the existence of three other specific free clinics for sexual and reproductive health problems, where all women regardless of being undocumented can attend.

As in the literature, in the thesis the lack of legal entitlement was widely considered a crucial barrier for immigrants to access healthcare services [17,36]. However, due to other individual and systemic factors, immigrants with different origins and social conditions have different access experiences, even under the same level of entitlement.

### Access

Related to access to healthcare, defined as the opportunity to reach and obtain appropriate healthcare services in situations of perceived need for care [27], it was found that information was only provided in the health centres and in the web page. Moreover, administrative professionals in health centres acted as a barrier to access healthcare when they were not fully aware of immigrants’ access rights or willing to offer information in an appropriate way. In addition, administrative processes to get access were considered difficult to navigate for immigrants [12,37]. The city council register was considered as one of the main obstacles to be granted access to care due to the high mobility of immigrants [17,37], being a prerequisite for getting the healthcare card. The fear of being billed also acted as a barrier on the decision to approach health centres [38].

Except for some gender-based conditions, the barriers which were related to immigrant women could also explain the difficulties of accessing healthcare services of immigrant men, such as the above-mentioned lack of entitlement or the influence of being undocumented. In the case of immigrant women, their autonomy and ability to reach healthcare were considered more limited for those forced into prostitution or experiencing other forms of gender-based violence [39,40]. Therefore, the scarcity of proactive research of the health issues among populations in situations of vulnerability was found to be a barrier to their access to healthcare services [8].

The language barrier was extensively described in the literature as in our studies as one of the main barriers for access [12,15,17,28,37,38,41]. In addition, it was found to also compromise the received biomedical care [41], affecting the appropriateness of the health services. Even if using translation tools is a possible strategy to overcome the language barrier, due to the high number of patients and a tight time to attend to them, healthcare professionals gave preference to concentrate on clinical aspects than to use such tools [18]. The use of cultural mediators could solve the lack of an effective communication [37], because using family members or untrained translators not only compromises the quality of care but also poses ethical conflicts about confidentiality [5].

Being undocumented was extensively considered as a great barrier for keeping good health and accessing healthcare services [14,16,38]. First, due to their documentation status and related social stigma from which they suffer [42]. Second, because of their lack of knowledge of their rights and of the functioning of the health system [15]. Third, for fearing rejection by health centre professionals or reported to immigration authorities when approaching health centres [8,9,12,15,17]. As facilitators for counteracting the fear, having social support and confidence towards public institutions were found, which was mainly related to having a large social network and a long time of residence in the host country [15,43].

Populations in situations of vulnerability tend to attend social-based resources and free clinics, where besides providing information and/or healthcare, they do advocacy work for their inclusion in the public health system. That is why free clinics and social organizations were presented as key resources on the promotion of the health and healthcare access of populations in situations of vulnerability [14,15,25]. However, the free clinics were also considered risky resources, for being parallel systems that may legitimize the inaction of the public health systems on immigrants’ health [14].

### Appropriateness

Considering that receiving appropriate care is also part of adequate access, in our results, the negative social consideration of immigrants and black people was considered to also played a role in access to healthcare services of Sub-Saharan African participants, as they perceived having received differentiated treatment in comparison to native people, so having felt discriminated against by professionals in the health centres [44]. Structural racism is rooted in the health system, its culture and norms and it also appears during the professional–patient interaction [45]. Even if the literature has repeatedly demonstrated that immigrants use healthcare services less than the native population [6,7,46,47], health centre staff may have the perception of the disproportionate use of healthcare services by immigrants [19]. This premise could result in increasing the access disparities, as especially in times of crisis, immigrants have been pointed out as ‘over users’ of healthcare services by society [48]. Moreover, it reinforces the social discourse that constructs migrants as ‘others’ who do not deserve healthcare and social benefits [28], creating ‘undesirable others’ based on racial and ethnic characteristics [49]. This othering process also maintains the power imbalance, including the entitlement to healthcare services [50].

In contrast to discriminatory practices, the right to health approach and healthcare professionals’ ethical guidelines promulgate the necessity of attending all patients equally, not discriminating against any person in the provision of healthcare.

### Methodological considerations and limitations of the studies

The studies contained certain methodological strengths and weaknesses that require mention. The used quantitative method, interrupted time series design, since it makes account for pre-intervention trends, is considered as a good method for assessing an intervention impact. However, further than the new policy implementation, other factors not taken into consideration in the study could also help explaining the results. Even if data from the public health system on the number of immigrants attended could have given a reliable picture and a more specific answer to the objective, unfortunately it was not publicly available.

The qualitative methods used allowed closer contact with the participants and captured essential characteristics of the topic, based on participants’ experience. In addition, the study design was flexible, as the focus of one study was based on the results of the previous one. All participants were purposely selected based on their knowledge and experience on the researched topic. Therefore, it was considered that their discourses could also represent the discourse of those who are in similar position. In addition, the study area and the recruitment process were widely explained in the thesis, letting the reader consider if the results could be transferred to their setting.

Related to credibility, as the author was at first not familiar with the study topic, she approached it with an open viewpoint that allowed considering the topic from a broader perspective than the experiential. However, the absence of familiarity with the study topic also posed some challenges, such as the possibility of failing to fully comprehend participants’ experiences or reflecting them properly.

The main limitation of the qualitative study considering the perception of the healthcare professionals was that even if the focus and the general aim of the study was put on the access of immigrant women, many of the participants’ answers included both immigrant men and women, even if they considered women as more vulnerable. Therefore, to better capture the influence of the gender-based factors when accessing healthcare services, the second qualitative study was only focused on women.

## Conclusions

Access to the Basque public health system for undocumented immigrants and immigrant women is subject to individual and systemic factors that may act as barriers or facilitators at different levels of access, namely entitlement, access, and appropriateness. For documented immigrants, the barriers for women and men were found to be similar, except for those women experiencing different forms of gender-based violence. Moreover, undocumented immigrants’ vulnerability led to fearing approaching health centres, so to encourage attendance at free clinics, which were considered to play a crucial role on providing healthcare attention and on doing advocacy on immigrants´ access.

Further than ensuring the legal entitlement of immigrants to healthcare, there is a need to provide them rights-based and culturally appropriate attention. In this regard, the use of cultural mediators, reinforcing non-discrimination values and the creation of more approachable and multilingual information sources are key aspects to improve all immigrants’ access and to get health systems that are organized for considering the needs of a culturally, linguistically, and socially diverse population.

## Paper context

Knowing that immigrants experience several barriers to access healthcare services, other factors of vulnerability such as being undocumented or suffering from gender based violence reinforced the difficulties of their access.

Among other individual and systemic factors, structural racism also influenced the willingness of health systems and staff to facilitate immigrants’ access. All considered, there is a need to reinforce rights based attention to get appropriate access to healthcare services for all.

## Does the health system respect immigrants’ right to health? Analysing immigrant women’s access to healthcare in the Basque Country (Spain)

Link to the full text of the PhD thesis:

http://hdl.handle.net/10810/33128
